# Perioperative Outcomes Following Single-Stage Surgery for Tandem Spinal Stenosis—A Single-Center Retrospective Cohort

**DOI:** 10.3390/jpm16070347

**Published:** 2026-06-26

**Authors:** Adham M. Khalafallah, Manav Daftari, Tanuj Prajapati, Sebastian Vargas-George, Anurag Aka, Christian K. Ramsoomair, Malek Bashti, Seth S. Tigchelaar, Timur Urakov

**Affiliations:** 1 Department of Neurological Surgery, University of Miami Miller School of Medicine, Miami, FL 33136, USA; manavdaftari@med.miami.edu (M.D.); tjp83@med.miami.edu (T.P.); sav74@miami.edu (S.V.-G.); asa350@miami.edu (A.A.); c.ramsoomair@med.miami.edu (C.K.R.); mbashti@uw.edu (M.B.); sst98@miami.edu (S.S.T.); turakov@med.miami.edu (T.U.); 2 Department of Neurological Surgery, University of Washington School of Medicine, Seattle, WA 98104, USA

**Keywords:** tandem spinal stenosis, single-stage surgery, perioperative outcomes, spine surgery, myelopathy

## Abstract

**Objectives**: Tandem spinal stenosis (TSS) is often underdiagnosed and traditionally managed with multi-stage surgery (MSS). Single-stage surgery (SSS) is an alternative, but prior studies largely emphasize younger, healthier patients. This study evaluated perioperative and functional outcomes after SSS for TSS in a surgically diverse cohort. **Methods**: A retrospective chart review included 20 patients who underwent SSS for TSS at a single academic institution. Mean age was 63.75 years, and median modified frailty index was 2. Etiologies included degenerative, traumatic, and neoplastic disease across cervical, thoracic, and lumbar regions. Outcomes included operative characteristics, complications, readmissions, and functional recovery measured by Visual Analog Scale (VAS) pain and modified Japanese Orthopaedic Association (mJOA) scores. **Results**: The mean number of operated levels was 5.2, mean operative time was 232.4 min, total OR time was 355.1 min, and length of stay was 6.9 days. Surgical complications occurred in 15% of patients, medical complications in 25%, and 90-day readmission in 15%, with no 30-day mortality. Mean mJOA improved from 12.86 at baseline to 16.08 at first follow-up and 16.46 at 3 months; REML mixed-effects modeling showed a significant timepoint effect (F (4, 34.55) = 9.15, *p* < 0.001), with significant Sidak-adjusted improvement at both timepoints. VAS pain showed no significant longitudinal effect. **Conclusions**: SSS for TSS appears feasible in a real-world, surgically diverse cohort including older and moderately frail patients. These findings support individualized SSS candidacy assessment.

## 1. Introduction

Tandem spinal stenosis (TSS), defined as pathological narrowing of the spinal canal at two or more non-contiguous spinal regions, is a rare and often overlooked diagnosis in patients with single-level spinal stenosis. TSS most commonly involves concurrent cervical and lumbar stenosis, though cervical–thoracic, thoracolumbar, and three-region combinations have been described. The prevalence of symptomatic TSS is estimated at approximately 10% in affected patient populations [1]. Clinically, patients with TSS may present with a complex constellation of symptoms, including gait disturbance, neurogenic claudication, myelopathy, and radiculopathy, making diagnosis and treatment particularly challenging. Because of this atypical presentation, diagnosis is often delayed, leading to worsening neurological deficits and poor treatment outcomes [1,2]. In a cadaveric study, Lee et al. found that cervical stenosis positively predicts concurrent lumbar stenosis 16.7% of the time and vice versa (15.3%), underscoring the importance of screening for multilevel disease when stenosis is identified in any single region [3].

The underlying etiology of tandem spinal stenosis is most commonly degenerative, including spondylotic changes and developmental canal stenosis [4,5]. Less common causes include trauma-related instability from motor vehicle collisions, falls, and spondylolisthesis, which can directly contribute to spinal stenosis [6,7]. Rarely, spinal tumors may also result in focal or multilevel canal narrowing due to mass effect or secondary degenerative changes [8,9,10]. Recognizing this etiological diversity is important, as it informs both the clinical presentation and surgical strategy for TSS.

When patients fail conservative treatment, surgical management of TSS is often necessary. The traditional surgical approach involves staged procedures, typically addressing the cervical region first, given the priority of myelopathy, followed by decompression of the remaining stenotic levels in a subsequent operation [4,5,6]. While staging allows for shorter individual operative sessions and may reduce the physiological burden of any single procedure, it requires multiple anesthetic exposures, separate hospitalizations, and prolonged cumulative recovery time, each of which carries its own risks and costs. In recent years, single-stage surgery (SSS), defined as decompression and/or stabilization of all affected spinal regions within a single operative session, has emerged as an alternative strategy. SSS was first described in 1987 by Dagi et al. in patients with concurrent cervical and lumbar stenosis [11]. The rationale for SSS centers on consolidating surgical intervention into a single anesthetic event and hospitalization, thereby eliminating the risks associated with a second operation, including repeat anesthesia, additional perioperative complications, and a second recovery period, while potentially reducing cumulative length of stay and overall healthcare costs. However, SSS entails longer operative and anesthesia times, greater intraoperative blood loss, and a more demanding immediate postoperative recovery, which must be weighed against the benefits of a single procedure. Several studies have reported comparable outcomes in functional recovery and complication rates between single-stage and multi-stage procedures; however, available studies remain limited and heterogeneous, and the literature regarding the safety and efficacy of SSS is limited [4,7,8].

Several gaps in the existing literature limit the ability to draw definitive conclusions regarding SSS. As highlighted by Ahorukomeye et al., prior studies have lacked standardized reporting of intraoperative parameters, often failing to distinguish total operating room time from surgical time, and have inconsistently reported estimated blood loss (EBL) [4,7,8]. Furthermore, many prior cohorts are composed of younger, healthier patients selected for SSS, introducing selection bias and limiting generalizability to older or frailer populations. Most reports also focus exclusively on degenerative cervical–lumbar stenosis, with limited data on TSS involving the thoracic spine or non-degenerative etiologies such as trauma or neoplasm. The purpose of this study is to report perioperative outcomes from a single-center cohort of patients who underwent SSS for TSS, with the aim of providing exploratory data on the feasibility of this approach in an older, moderately frail, and etiologically diverse patient population.

## 2. Materials and Methods

### 2.1. Patient Population and Selection

A retrospective chart review was conducted at a single academic tertiary-care center, following Institutional Review Board approval, for cases performed between July 2020 and September 2024. For this study, TSS was defined as radiographic stenosis involving two or more non-contiguous spinal regions. Included patients underwent SSS involving two or more spinal levels for TSS secondary to underlying pathology, including degenerative disk disease, traumatic instability, or neoplasm. Patients were identified through operative logs and confirmed by review of preoperative imaging demonstrating stenosis at two or more non-contiguous spinal regions. The decision to pursue SSS rather than staged surgery was made at the discretion of the treating surgeon based on clinical factors including symptom severity, anatomical distribution of stenosis, medical comorbidities, and anticipated tolerance of a single prolonged operative session. No formal protocol or algorithm was used to assign patients to SSS versus staged surgery during the study period. Patients with TSS who underwent staged procedures during this period were not captured in this cohort, and therefore no direct comparison group is available.

### 2.2. Outcome Measures

Information on patient demographics, medical history, surgical characteristics, and perioperative outcomes was collected. Intraoperative variables recorded included the number of spinal levels operated, frequency of operated spinal regions, frequency of procedure type, operative time, and estimated blood loss (EBL). Operative time (OT) was defined as the duration from skin incision to skin closure. Total operating room (TOR) time encompassed the entire period the patient remained in the OR, including anesthesia induction, positioning, surgical procedure, and emergence from anesthesia.

Postoperative outcomes were categorized into complications and recovery metrics. Complications included surgical complications, medical complications, 90-day readmission rates, and reoperation rates. Surgical complications were defined as adverse events directly attributable to the surgical procedure, as assessed by the primary care team. Medical complications were defined as postoperative issues arising from pre-existing medical comorbidities. Readmissions were tracked for up to 90 days postoperatively.

Recovery was evaluated using the Visual Analog Scale (VAS) and the modified Japanese Orthopaedic Association (mJOA) score [9,10,12]. No single validated outcome measure comprehensively captures the full clinical heterogeneity of this cohort. The mJOA was therefore selected as the most clinically applicable available tool, given the predominance of cervical involvement in our cohort. VAS and mJOA scores were assessed preoperatively and at postoperative follow-up intervals: initial follow-up, 3 months, 6 months, and 12 months.

### 2.3. Statistical Analysis

Descriptive statistics are reported as frequencies and percentages for categorical variables and as mean (standard deviation) for continuous variables, unless otherwise specified. Medians and ranges are reported where clinically relevant. Etiology-based subgroup summaries were descriptive only and were not tested inferentially because subgroup sizes were small.

Longitudinal VAS pain and mJOA total scores were analyzed using separate linear mixed-effects models estimated by restricted maximum likelihood. For each outcome, timepoint was modeled as a categorical fixed effect and patient was modeled as a random intercept to account for within-patient correlation across repeated measurements. Denominator degrees of freedom and *p*-values for fixed effects were estimated using the Satterthwaite approximation. The omnibus timepoint effect was evaluated using a Type III F-test, with a likelihood-ratio test performed as a secondary model comparison after refitting full and intercept-only models with maximum likelihood. Estimated marginal means and 95% confidence intervals were calculated at each timepoint. Prespecified postoperative-versus-baseline contrasts were adjusted using the Sidak method. For paired baseline and postoperative observations, Hedges’ g_av with small-sample correction was calculated as a standardized effect size. Statistical analyses were performed in R version 4.5.1 using lme4 version 1.1.37, ggplot2 version 3.5.2, and dplyr version 1.1.4.. A two-sided *p*-value < 0.05 was considered statistically significant. All analyses are hypothesis-generating, given the single-center retrospective design and small sample size.

### 2.4. Literature Review

A narrative review of the literature was performed to contextualize the present findings within the evidence on single-stage surgery for TSS. Articles published between 1986 and 2025 were identified by searching PubMed using the following search terms and iterations: “spine surgery”, “staged surgery”, “single-stage”, “postoperative outcomes”, and “complications.” This review was not intended to be systematic; rather, it provides a narrative summary of the most relevant comparative studies.

Each study was then reviewed based on the following inclusion and exclusion criteria. Studies were included based on the following criteria: (1) original research articles, (2) full-text availability, and (3) inclusion of patients who underwent either single-stage or multi-stage spinal procedures. Studies focusing on pediatric populations or investigating a single specific pathology (e.g., congenital abnormalities) were excluded.

## 3. Results

### 3.1. Patient Demographics

A total of 20 patients underwent SSS, including 13 males and 7 females, with a mean age of 63.75 years. The racial distribution of the cohort included White Non-Hispanic (*n* = 7), White Hispanic (*n* = 8), and African American (*n* = 5) individuals. A review of preoperative medical history revealed that the most common comorbidities were hypertension (55%), diabetes mellitus (20%), heart disease (20%), hyperlipidemia (30%), and chronic kidney disease (5%). The modified five-item frailty index (mFI-5) was calculated for each patient. Of patients included in this study, the mean mFI-5 was 1.74, and the median was 2.0 [13,14]. The mean body mass index (BMI) of the study population was 27.8 kg/m^2^ (Table 1).

### 3.2. Intraoperative Outcomes

**Figure 1:**
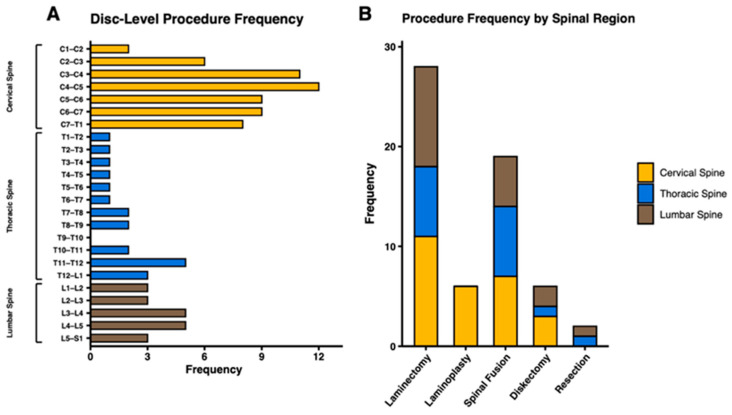
Intraoperative data demonstrating (**A**) frequency of operated levels and (**B**) frequency of procedures performed.

Among all spinal levels, the C4–C5 segment was the most frequently operated level (12.1%; Figure 1A). Overall, the mean number of operated spinal levels was 5.15 per case. The cervical region accounted for the greatest operative burden, with a mean of 3.1 operated levels per case, followed by the thoracic and lumbar regions, with means of 1.05 and 1.0 operated levels per case, respectively. The most commonly performed procedure was laminectomy (*n* = 28), followed by fusion procedures (*n* = 18), including anterior cervical discectomy and fusion (ACDF), transforaminal lumbar interbody fusion (TLIF), and anterior lumbar interbody fusion (ALIF). Other procedures included laminoplasty (*n* = 6), discectomy (*n* = 6), and lesion resection (*n* = 2). One patient underwent resection of an arachnoid cyst, while another underwent resection of thoracic and lumbar extramedullary tumors (Figure 1B).

The most common anatomic combination was cervical–lumbar disease (*n* = 10), followed by cervical–thoracic disease (*n* = 7) and thoracic–lumbar disease (*n* = 3). No patients underwent three-region cervical–thoracic–lumbar surgery. Same-setting procedure combinations were grouped into decompression-only, decompression with fusion, and discectomy/fusion-based procedures. Among cervical–lumbar cases, five patients underwent laminectomy- or laminoplasty-based decompression-only combinations, four underwent procedures that included fusion or discectomy/fusion, and one underwent cervical discectomy with lumbar hemilaminectomy. Among cervical–thoracic cases, procedures primarily consisted of cervical decompression and/or fusion paired with thoracic decompression and/or fusion. All thoracic–lumbar cases involved thoracic laminectomy with lumbar laminectomy.

The mean operative time was 232.4 min, with a mean TOR time of 355.1 min. The mean EBL was 234.3 mL. Following surgery, patients had an average hospital LOS of 6.9 days. In a subgroup analysis excluding two patients who underwent surgery due to trauma, the average LOS decreased to 4.98 days (Table 2).

Descriptive subgroup summaries by etiology are provided in Table S1. Most patients had degenerative pathology (*n* = 16, 80%), while traumatic and lesion-related pathology each accounted for two patients (10%). Trauma cases had longer hospital length of stay than the overall cohort, with a mean LOS of 24.2 days, compared with 4.7 days in degenerative cases and 7.3 days in lesion-related cases. Mean EBL was 208.4 mL in degenerative cases, 275.0 mL in traumatic cases, and 400.0 mL in lesion-related cases. These subgroup summaries were descriptive only and were not tested inferentially because of the small subgroup sizes.

### 3.3. Postoperative Outcomes

Three patients (15%) experienced surgical complications directly attributable to the surgical procedure: one patient developed a rare titanium allergy, and two patients developed surgical site infections (SSI) requiring readmission. The patient with the titanium allergy presented with recurrent neck swelling and lymphadenopathy. A comprehensive infectious and allergy workup confirmed an allergy to titanium. Although the patient’s myelopathic symptoms improved, revision spine surgery was ultimately required for removal of the titanium hardware due to persistent swelling and lymphadenopathy. Both patients with SSI were successfully managed with wound washout. These wound washouts were counted as surgical complications and readmissions but were not included in the revision spine surgery count. In total, two patients underwent revision spine surgery: the patient requiring hardware removal for titanium allergy and one additional patient who underwent revision lumbar fusion for progressive spondylosis refractory to conservative management.

Five patients (25%) developed postoperative medical complications related to pre-existing comorbidities or hospitalization. Two patients were admitted for trauma-related injuries and sustained postoperative complications attributable to their trauma, including acute respiratory distress syndrome (ARDS) and right upper extremity edema. Two patients experienced rapid ventricular response from underlying atrial fibrillation that was successfully treated. One patient experienced dysphasia, unrelated to the surgery (Table 3). The mean duration of postoperative follow-up was 8.21 months. Three patients were lost to follow-up at their first postoperative visit. Three patients (15%) were readmitted to the hospital within 90 days postoperatively. The mortality rate following TSS surgery was zero when assessed 30 days postoperatively (Table 3).

**Figure 2:**
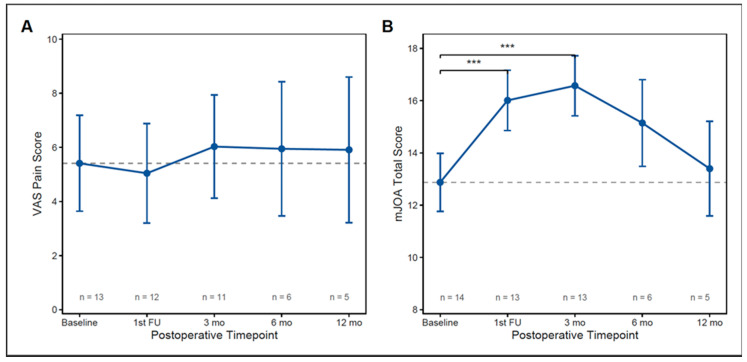
Longitudinal clinical outcome scores after single-stage surgery for tandem spinal stenosis. (**A**) VAS pain scores and (**B**) mJOA total scores are shown as estimated marginal means with 95% confidence intervals from restricted maximum likelihood linear mixed effects. Dashed horizontal lines indicate baseline estimated marginal means. Brackets denote prespecified postoperative-versus-baseline contrasts that remained significant after Sidak adjustment (*** *p* < 0.001).

Preoperatively, patients reported a mean VAS pain score of 5.31 ± 3.71, consistent with a moderate amount of pain. Mean VAS scores were 5.17 ± 3.46 at first follow-up, 6.36 ± 1.96 at 3 months, 6.00 ± 3.63 at 6 months, and 5.40 ± 2.07 at 12 months. In the REML linear mixed-effects model, there was no significant omnibus effect of timepoint on VAS pain scores (F (4, 29.39) = 0.26, *p* = 0.901), and no postoperative-versus-baseline contrast remained significant after Sidak adjustment (Figure 2A).

### 3.4. Literature Review

The narrative review returned 171 entries, of which 8 were reviewed in detail. Systematic and literature reviews and studies that did not directly compare single-stage and multi-stage procedures were excluded. The studies summarized in Table 4 examined single-stage surgical approaches for spine procedures involving non-contiguous spinal segments.

## 4. Discussion

Single-stage surgery enables simultaneous decompression of multiple affected spinal regions within a single operative session. Given the potential for perioperative complications in spine surgery, selecting the optimal surgical strategy for patients with TSS requires careful consideration [21]. In this study, we report perioperative outcomes from a cohort of 20 patients who underwent SSS for TSS at a single academic tertiary-care center. Our findings provide exploratory, hypothesis-generating data on the perioperative characteristics of SSS in a clinical context that has been underrepresented in prior literature. First, we report detailed intraoperative parameters, including discrete measurements of anesthesia and operative time, EBL, postoperative complications, and hospital LOS, allowing for a more granular assessment of surgical efficiency. Second, we extend the evidence base for SSS into a patient profile that has not been fully characterized in prior literature: a cohort that is older, moderately frail, etiologically heterogeneous, and anatomically diverse (incorporating a range of spinal procedures across multiple regions). However, this etiological and procedural diversity, while reflective of real-world practice, also limits the ability to attribute outcomes to any specific pathology or anatomical combination, and pooled results should be interpreted accordingly. Third, by demonstrating preliminary feasibility and acceptable short-term complication rates, these findings may inform future prospective studies aimed at refining patient selection criteria and evaluating the comparative effectiveness of SSS versus staged approaches.

The procedural and anatomical diversity represented in this study underscores the potential applicability of SSS across varied surgical contexts, though this remains to be confirmed in larger controlled studies. To our knowledge, this is the first SSS series to span all three spinal regions, including the thoracic spine, while encompassing degenerative, traumatic, and neoplastic etiologies in a cohort with mean age and frailty scores meaningfully higher than those reported in prior SSS cohorts. To contextualize these findings, we compare our results with previously reported outcomes from studies evaluating single-stage and multi-stage surgical approaches. However, all comparisons are informal and cross-study in nature, as this cohort lacks an internal comparator group. Differences in patient populations, surgical techniques, outcome definitions, and follow-up periods across studies preclude direct or causal comparisons.

### 4.1. Patient Selection

Careful patient selection remains crucial for optimizing outcomes in SSS. Prior studies suggest that SSS may be most appropriate for patients without severe medical comorbidities, age younger than 60 years, and no contraindications to surgery [18]. Furthermore, patients with adult spinal deformity, increased frailty, moderate-to-severe pelvic incidence–lumbar lordosis mismatch, or a higher number of levels requiring surgery may benefit from multi-stage approaches. Despite longer operative times and hospital stays, MSS may mitigate perioperative risks in select cases. In this cohort, SSS was performed in an older population with moderate frailty, with surgical complications in 15% of patients, medical complications in 25%, 90-day readmission rate in 15%. There was no observed 30-day mortality, and patients demonstrated early improvement in myelopathic symptoms. These findings do not establish that SSS is appropriate for all TSS patients. Rather, they raise the hypothesis that age, moderate frailty, non-degenerative etiology, and thoracic involvement may not, in isolation, serve as exclusion criteria for SSS consideration. From a personalized surgical care perspective, these findings support conceptualizing SSS candidacy as a multidimensional, individualized assessment. Further studies are warranted to clarify preoperative risk factors and to guide optimal surgical decision-making between SSS and MSS.

### 4.2. Intraoperative Outcomes

Intraoperative complications during spinal surgery can include anesthesia-related events, vascular injury, and neurologic injury. A retrospective study of 294 adult patients reported an intraoperative adverse event rate of 6.9%, classified by the Spine Adverse Events Severity System version 2 (SAVES-V2) [22,23]. Sun et al. demonstrated that SSS in 51 TSS patients reduced operative time compared to staged approaches [18]. Similar findings have been reported across multiple comparative studies [4,19]. Our cohort aligns with these reports, showing a mean operative time of 232.4 min. Notably, prior studies have not consistently distinguished between TOR and OT, potentially overestimating the procedural burden of SSS [4]. In this cohort, we separately quantified TOR and OT, revealing a 122 min difference and enabling a more accurate evaluation of intraoperative efficiency.

However, the absolute operative and total OR times in this cohort are substantial (mean OT 232.4 min, mean TOR 355.1 min), reflecting the complexity of multilevel, multiregional procedures. In the context of contemporary trends toward shorter operative sessions, ambulatory surgery center models, and enhanced recovery pathways, these prolonged times represent a significant facility-level resource commitment. Whether the consolidation of two separate admissions, anesthetic events, and recovery periods into a single episode of care offsets the increased per-case resource utilization remains an important question. In bundled payment or DRG-based reimbursement systems, the financial implications of a single prolonged hospitalization versus two shorter admissions warrant formal cost-effectiveness analysis. The feasibility of SSS may therefore be most practical in academic or tertiary-care settings with the infrastructure, multidisciplinary support, and operative scheduling flexibility to accommodate these cases.

Minimizing intraoperative time is critical for reducing postoperative complications. Recent analyses demonstrate that prolonged operative duration is independently associated with an increased risk of both overall and medical complications following surgery [24,25]. Therefore, limiting intraoperative time while maintaining surgical efficacy is a key factor in optimizing patient outcomes.

Another major intraoperative concern is vascular injury resulting in excessive blood loss. The mean EBL in our cohort was 234.3 mL, consistent with the expected blood loss during decompression procedures [26,27]. On average, patients underwent multiple procedure types across more than five levels, findings consistent with other comparative studies of SSS and MSS for TSS [18,19]. Notably, increased blood loss not only poses intraoperative risks but also raises the need for transfusion, which has been linked to longer ICU stays and increased risk of transfusion-related complications [27,28].

Lastly, the average LOS in our cohort was 6.9 days. This overestimates average LOS due to the inclusion of two patients who underwent SSS for trauma. In a subgroup analysis excluding these patients, average LOS decreased to 4.98 days. The influence of these two trauma patients on overall LOS highlights the impact of cohort heterogeneity on pooled outcomes; formal sensitivity analyses across etiologic subgroups would strengthen the interpretability of these findings. Nevertheless, our LOS findings align with comparative studies demonstrating shorter LOS for patients undergoing SSS compared to staged approaches [18,19]. In a retrospective study of 51 patients, Sun et al. reported notable differences in mean LOS between SSS (16.6 days) and multi-stage surgery (MSS), with the first cervical-first cohort averaging 22.75 days and the lumbar-first surgery cohort averaging 22.89 days [18]. These findings were further supported by a systematic review and meta-analysis by Lu et al., which also demonstrated a significant reduction in LOS for SSS compared to MSS [8].

### 4.3. Postoperative Complications

Postoperatively, surgical complications occurred in three patients (15%), with no 30-day mortality. All surgical complications were effectively managed through either infectious disease management or revision surgery. Medical complications following SSS were observed in five patients (25%). After excluding patients admitted for trauma-related injuries, the rate of complications directly attributable to SSS decreased to 15%. All medical complications were successfully managed postoperatively, with no associated mortality. In cross-study comparisons, which should be interpreted cautiously given differences in populations and methodology, prior reports have described similar complication rates after SSS [18,19]. Additionally, Albayar et al. reported significantly lower rates of deep vein thrombosis (DVT) and pulmonary embolism (PE) in single-stage procedures, even after adjusting for comorbidities and extent of fusion (≥4 levels) [17]. Edwards et al. found an 8.17-fold increased risk of DVT/PE in multi-stage procedures, despite lower preoperative risk in that group [20]. These findings are particularly important given the long-term risks: five-year mortality rates of 25% for DVT and 50–60% for PE [29]. No patients in this cohort experienced DVT or PE, although this finding should be interpreted cautiously given the small sample size (*n* = 20).

### 4.4. Functional Recovery

Patients in this cohort demonstrated significant improvement in myelopathic symptoms postoperatively, as assessed by mJOA scores. The mJOA was originally validated for cervical myelopathy, and its application to patients with concurrent thoracic or lumbar pathology in this cohort represents an extrapolation that should be considered when interpreting these results [12]. Clinical improvement was observed up to six months postoperatively when compared to baseline scores. Statistically significant improvements were observed at both the first postoperative visit (mean difference 3.13, Hedges’ g_av = 1.16, *p* < 0.001) and at three months (mean difference 3.70, Hedges’ g_av = 1.75, *p* < 0.001), representing large effect sizes. Sustained clinical gains beyond this period were less apparent, with available observations declining from 14 patients at baseline to 5 at 12 months. The apparent return of mJOA scores toward preoperative levels at 12 months should be interpreted with caution, as this pattern likely reflects differential attrition rather than true clinical deterioration. Patients who experienced meaningful neurological improvement may have been less likely to maintain follow-up, leaving a subset of patients with persistent or recurrent symptoms overrepresented at later timepoints. The linear mixed-effects model accounts for unbalanced data by using all available observations rather than restricting analysis to complete cases, which mitigates but does not eliminate the potential for attrition bias. Sun et al. reported more than a 200% improvement in myelopathic symptoms, measured by JOA-C and JOA-L scores, in patients undergoing SSS; however, no significant differences were found in intergroup analysis [18]. In a separate study of 13 patients undergoing single-stage decompression and fusion in cervical and lumbar regions, significant intragroup improvement in myelopathic symptoms was noted postoperatively. Interestingly, contrary to previous reports, a significant difference favoring MSS was found in intergroup comparisons [19]. Taken together, these findings suggest that SSS may be associated with meaningful short-term functional improvement, though the comparative effectiveness relative to MSS remains uncertain and requires prospective, controlled investigation.

In contrast to mJOA scores, VAS pain scores demonstrated a trend toward worsening rather than improvement postoperatively. The presence of increased or persistent pain may reflect multiple factors inherent to SSS. Alvin et al. reported that cervical surgery significantly improved VAS neck and arm pain at one year, but neither cervical nor lumbar decompression significantly improved low back pain in 84 TSS patients [30]. Inoue et al. similarly demonstrated that although 69% of TSS patients in their cohort initially showed improved lumbar symptoms following cervical decompression alone, only 22% maintained that improvement, with the majority having symptoms relapse [31]. Additionally, the small sample size of our cohort may also have amplified the role of patient-specific factors that affected the observed pain scores. These findings highlight the need for multimodal perioperative pain strategies in this population.

### 4.5. Limitations

This study has several limitations that must be considered. First, the retrospective nature of data collection limits the completeness of available information, as analysis relied on existing medical records. Second, the small sample size (*n* = 20) limits the statistical power and generalizability of the findings, particularly when estimating complication rates and functional outcomes; accordingly, all findings should be considered exploratory and hypothesis-generating rather than confirmatory. Third, the cohort included patients with diverse underlying etiologies and a range of spinal procedures across multiple regions. While this reflects the heterogeneous clinical scenarios encountered in TSS management, pooling degenerative, traumatic, and neoplastic etiologies with markedly different procedures (e.g., ACDF, TLIF, laminoplasty, tumor resection) creates a clinically heterogeneous cohort that complicates the interpretation of aggregate outcomes. Fourth, patient selection for single-stage surgery reflected surgeon judgment and institutional practice patterns, introducing potential selection bias in determining which patients were considered appropriate candidates for SSS. The absence of data on patients with TSS who underwent staged procedures during the study period further limits the ability to assess selection effects. Fifth, the absence of a direct comparative group undergoing MSS restricts the conclusions that can be drawn regarding the relative benefit of SSS. Comparisons were instead drawn from previously published data, which may differ in patient demographics, surgical techniques, and perioperative care protocols. Sixth, follow-up attrition was substantial, with available observations declining from 14 patients at baseline to 5 at 12 months. Future prospective studies with larger sample sizes and standardized follow-up protocols are warranted to further evaluate the safety and efficacy of SSS for TSS.

## 5. Conclusions

This single-center retrospective cohort of 20 patients provides exploratory data suggesting that single-stage surgery for tandem spinal stenosis may be feasible in selected patients, including those of advanced age and moderate frailty, with acceptable short-term complication rates and no perioperative mortality. Significant improvement in myelopathic symptoms was observed at early postoperative timepoints, though interpretation of longer-term outcomes is limited by substantial follow-up attrition. The retrospective design, small sample size, heterogeneous cohort, and absence of an internal comparator group preclude conclusions about the comparative safety or effectiveness of SSS relative to staged surgery. These findings are hypothesis-generating and should be interpreted within the context of a single academic tertiary-care center. Future prospective multicenter studies with larger sample sizes, standardized patient selection criteria, and longer follow-up are needed to define the role of SSS in the surgical management of TSS.

## Figures and Tables

**Table 1:** Demographic and clinical characteristics of the study population.

Variable	Value (*N* = 20)
Age in years, mean [range]	63.75 [40–82]
Sex, female, *n* (%)	7 (35%)
Race, *n* (%)	
White (non-Hispanic)	7 (35%)
White (Hispanic)	8 (40%)
African American	5 (25%)
Smoking History, *n* (%)	9 (45%)
Modified 5-Item Frailty Index (mFI-5)	
Mean (SD)	1.74 (1.28)
Median	2.0
Comorbidities, *n* (%)	
Hypertension	11 (55%)
Diabetes Mellitus	4 (20%)
Heart Disease	4 (20%)
Chronic Kidney Disease	1 (5%)
Hyperlipidemia	6 (30%)
Body Mass Index (BMI), mean (SD)	27.83 (4.28)

**Table 2:** Intraoperative outcomes of patients undergoing SSS.

Variable	Value (N = 20)
Estimated Blood Loss (EBL) in mL, mean (SD)	234.25 (237.4)
Mean Total Operating Room Time, mean (SD)	355.05 (122.0)
Mean Operative Time in minutes, mean (SD)	232.42 (98.3)
Length-of-Stay in days, mean (SD)	6.90 (6.8)
Total Surgical Levels (All Regions), *n*	103
Cervical	62
Thoracic	21
Lumbar	20
Total Mean Operated Levels, mean	5.15
Cervical	3.1
Thoracic	1.05
Lumbar	1.0

**Table 3:** Postoperative outcomes following SSS. Values are n (%) with 95% Wilson score confidence intervals for proportions.

Variable	Value (N = 20)	95% CI
Surgical Complications, *n* (%)	3 (15%)	5.2–36.0%
Medical Complications, *n* (%)	5 (25%)	11.2–46.9%
Atrial Fibrillation with RVR	2 (10%)	2.8–30.1%
Right Upper Extremity Edema	1 (5%)	0.9–23.6%
ARDS	1 (5%)	0.9–23.6%
Dysphasia	1 (5%)	0.9–23.6%
Re-Admission at 90 Days Post-op, *n* (%)	3 (15%)	5.2–36.0%
Re-Operations, *n*	2 (10%)	2.8–30.1%
Mean Follow-up in months, mean [range]	8.21 [0.07–35.93]	--
Lost to Follow Up, *n*	3	
30-day Mortality (%)	0	0.0–16.1%

**Table 4:** Summary of literature review results.

Citation	Title	Study Aim	Findings
Passias et al. 2024 [15]	When is staging complex adult spinal deformity advantageous? Identifying subsets of patients who benefit from staged interventions	Compare outcomes in multi-stage versus single-stage combined-approach surgeries based on patient and surgical factors	Although outcomes were comparable overall, patients with increased frailty, PI-LL mismatch, and higher anticipated levels fused benefited more from multi-staged procedures due to fewer perioperative complications.
Illescas et al. 2024 [16]	A Nationwide Comparison of Outcomes and Resource Use in Staged vs. Simultaneous Cervical and Lumbar Fusions: A Retrospective Database Study	Compare postoperative complications and resource use in multi-stage versus single-stage cervical and lumbar fusions	Simultaneous fusion was associated with higher rates of ICU admission and prolonged hospital stay compared to multi-staged fusion.
Albayar et al. 2023 [17]	Comparison of Staged vs. Same-Day Circumferential Spinal Fusions for Adult Spinal Deformity (ASD)	Compare perioperative outcomes and costs of multi-stage versus single-stage circumferential ASD corrective surgeries	MSS was associated with significantly increased length of stay, risk of pulmonary embolism, and admission costs compared to SSS.
Sun et al. 2021 [18]	Simultaneous or Staged Decompressions for Patients with Tandem Spinal Stenosis	Compare the clinical effects of cervical decompression first, lumbar decompression first, or simultaneous decompression of both lesions in the treatment of TSS	Simultaneous decompression was associated with less blood loss and significantly shorter operative time and hospital stay compared to multi-staged cervical or lumbar decompression.
Cao et al. 2021 [19]	Simultaneous or staged operation for tandem spinal stenosis: surgical strategy and efficacy comparison	Compare the efficacy of MSS versus SSS for patients with TSS	Simultaneous decompression was associated with shorter hospitalization time without an increase in operative time or bleeding, indicating similar safety and efficacy to multi-staged procedures.
Edwards et al. 2018 [20]	Deep Vein Thrombosis After Complex Posterior Spine Surgery: Does Staged Surgery Make a Difference?	Compare the incidence of deep vein thrombosis (DVT) in single- versus multi-stage posterior-only complex spinal surgeries	MSS was associated with a higher risk of DVT compared to single-stage procedures.

## Data Availability

The data are not publicly available due to patient privacy and institutional restrictions in accordance with HIPAA regulations.

## Supplementary Materials

The following supporting information can be downloaded at https://www.mdpi.com/article/10.3390/jpm16070347/s1, Table S1: Descriptive perioperative outcomes stratified by etiologic subgroup.

## Abbreviations

The following abbreviations are used in this manuscript:

TSS	Tandem spinal stenosis
MSS	Multi-stage surgery
SSS	Single-stage surgery
mJOA	modified Japanese Orthopaedic Association
VAS	Visual Analog Scale
mFI-5	Modified 5-item frailty index
BMI	Body mass index
EBL	Estimated blood loss
TOR	Total operating room time
OT	Operative time
LOS	Length of stay
SSI	Surgical site infection
ARDS	Acute respiratory distress syndrome
ACDF	Anterior cervical discectomy and fusion
TLIF	Transforaminal lumbar interbody fusion
ALIF	Anterior lumbar interbody fusion
SAVES-V2	Spine Adverse Events Severity System version 2
DVT	Deep vein thrombosis
PE	Pulmonary embolism
ICU	Intensive care unit
JOA-C	Japanese Orthopaedic Association cervical score
JOA-L	Japanese Orthopaedic Association lumbar score
REML	Restricted maximum likelihood
CI	Confidence interval

## References

[1] Baker J.F. (2020). Evaluation and Treatment of Tandem Spinal Stenosis. J. Am. Acad. Orthop. Surg..

[2] Overley S.C., Kim J.S., Gogel B.A., Merrill R.K., Hecht A.C. (2017). Tandem Spinal Stenosis: A Systematic Review. JBJS Rev..

[3] Lee M.J., Garcia R., Cassinelli E.H., Furey C., Riew K.D. (2008). Tandem stenosis: A cadaveric study in osseous morphology. Spine J..

[4] Ahorukomeye P., Saniei S., Pennacchio C.A., Kuo A., Mlis A.C.S., Cheng C.W., Furey C.G. (2022). Outcomes in surgical treatment for tandem spinal stenosis: Systematic literature review. Spine J..

[5] Jannelli G., Baticam N.S., Tizi K., Truffert A., Lascano A.M., Tessitore E. (2020). Symptomatic tandem spinal stenosis: A clinical, diagnostic, and surgical challenge. Neurosurg. Rev..

[6] Luo C.A., Kaliya-Perumal A.K., Lu M.L., Chen L.H., Chen W.J., Niu C.C. (2019). Staged surgery for tandem cervical and lumbar spinal stenosis: Which should be treated first?. Eur. Spine J..

[7] Mittal S., Ahuja K., Sudhakar P.V., Ifthekar S., Yadav G., Sarkar B., Kandwal P. (2022). Simultaneous decompression of all stenotic regions versus decompression of only the most symptomatic region in patients with tandem spinal stenosis: A systematic review and meta-analysis. Eur. Spine J..

[8] Lu C., Qiu H., Huang X., Yang X., Liu D., Zhang S., Zhang Y. (2023). Meta-Analysis of Simultaneous versus Staged Decompression of Stenotic Regions in Patients with Tandem Spinal Stenosis. World Neurosurg..

[9] Delgado D.A., Lambert B.S., Boutris N., McCulloch P.C., Robbins A.B., Moreno M.R., Harris J.D. (2018). Validation of Digital Visual Analog Scale Pain Scoring With a Traditional Paper-based Visual Analog Scale in Adults. J. Am. Acad. Orthop. Surg. Glob. Res. Rev..

[10] Yamada T., Yoshii T., Yamamoto N., Hirai T., Inose H., Okawa A. (2018). Surgical outcomes for lumbar spinal canal stenosis with coexisting cervical stenosis (tandem spinal stenosis): A retrospective analysis of 565 cases. J. Orthop. Surg. Res..

[11] Dagi T.F., Tarkington M.A., Leech J.J. (1987). Tandem lumbar and cervical spinal stenosis. Natural history, prognostic indices, and results after surgical decompression. J. Neurosurg..

[12] Tetreault L., Kopjar B., Nouri A., Arnold P., Barbagallo G., Bartels R., Qiang Z., Singh A., Zileli M., Vaccaro A. (2017). The modified Japanese Orthopaedic Association scale: Establishing criteria for mild, moderate and severe impairment in patients with degenerative cervical myelopathy. Eur. Spine J..

[13] Weaver D.J., Malik A.T., Jain N., Yu E., Kim J., Khan S.N. (2019). The Modified 5-Item Frailty Index: A Concise and Useful Tool for Assessing the Impact of Frailty on Postoperative Morbidity Following Elective Posterior Lumbar Fusions. World Neurosurg..

[14] Lucido T., Rajkumar S., Rogowski B., Meinert J., Elhamdani S., Liang Y., Karlovits S., Yu A., Wegner R.E., Shepard M.J. (2024). The 5-factor modified frailty index as a prognostic factor of stereotactic radiosurgery for metastatic disease to the brain. J. Neurosurg..

[15] Passias P.G., Tretiakov P., Onafowokan O.O., Das A., Lafage R., Line B.G., Nayak P., Diebo B., Daniels A.H., Gum J.L. (2025). When is staging complex adult spinal deformity advantageous? Identifying subsets of patients who benefit from staged interventions. J. Neurosurg. Spine.

[16] Illescas A., Poeran J., Zhong H., Cozowicz C., Girardi F.P., Memtsoudis S.G., Liu J. (2024). A Nationwide Comparison of Outcomes and Resource Use in Staged vs Simultaneous Cervical and Lumbar Fusions: A Retrospective Database Study. HSS J..

[17] Albayar A., Santangelo G., Spadola M., Macaluso D., Ali Z.S., Saifi C., Heintz J., Han X., Bilker W., Malhotra N. (2023). Comparison of Staged vs Same-Day Circumferential Spinal Fusions for Adult Spinal Deformity. Int. J. Spine Surg..

[18] Sun W.Z., Yan X., Yang Y.L., Song H., Xia Z., Yang S., Chen F., Li W., Yu Z., Liu B. (2021). Simultaneous or Staged Decompressions for Patients with Tandem Spinal Stenosis. Orthop. Surg..

[19] Cao J., Gao X., Yang Y., Lei T., Shen Y., Wang L., Tian Z. (2021). Simultaneous or staged operation for tandem spinal stenosis: Surgical strategy and efficacy comparison. J. Orthop. Surg. Res..

[20] Edwards C.C., Lessing N.L., Ford L., Edwards C.C. (2018). Deep Vein Thrombosis After Complex Posterior Spine Surgery: Does Staged Surgery Make a Difference?. Spine Deform.

[21] Lange N., Stadtmüller T., Scheibel S., Reischer G., Wagner A., Meyer B., Gempt J. (2022). Analysis of risk factors for perioperative complications in spine surgery. Sci. Rep..

[22] Lovi A., Gallazzi E., Galbusera F., Colombini A., Pregliasco F., Peretti G., Brayda-Bruno M. (2022). Perioperative adverse events in adult and pediatric spine surgery: A prospective cohort analysis of 364 consecutive patients. Brain Spine.

[23] Rampersaud Y.R., Anderson P.A., Dimar J.R., Fisher C.G. (2016). Spine Trauma Study Group; Degenerative Spine Study Group. Spinal Adverse Events Severity System, version 2 (SAVES-V2): Inter- and intraobserver reliability assessment. J. Neurosurg. Spine.

[24] Findlay M.C., Kim R.B., Warner W.S., Sherrod B.A., Park S., Mazur M.D., Mahan M.A. (2024). Identification of an Operative Time Threshold for Substantially Increased Postoperative Complications Among Elderly Spine Surgery Patients. Glob. Spine J..

[25] Monetta A., Griffoni C., Falzetti L., Evangelisti G., Noli L.E., Tedesco G., Cavallari C., Bandiera S., Terzi S., Ghermandi R. (2024). Prolonged operative time significantly impacts on the incidence of complications in spinal surgery. J. Orthop. Surg. Res..

[26] Inose H., Kato T., Yuasa M., Yamada T., Maehara H., Hirai T., Yoshii T., Kawabata S., Okawa A. (2018). Comparison of Decompression, Decompression Plus Fusion, and Decompression Plus Stabilization for Degenerative Spondylolisthesis: A Prospective, Randomized Study. Clin. Spine Surg..

[27] Hu S.S. (2004). Blood loss in adult spinal surgery. Eur. Spine J..

[28] Chen J.W., Chanbour H., Roth S.G., Stephens B.F., Abtahi A.M., Zuckerman S.L. (2023). How Much Blood Loss Is Appropriate for a 2- to 3-Level Posterior Lumbar Fusion?. Int. J. Spine Surg..

[29] Amaral C., Guimaraes Pereira L., Moreto A., Sa A.C., Azevedo A. (2017). The postoperative venous thromboembolism (TREVO) study—Risk and case mortality by surgical specialty (Estudo TRomboEmbolismo Venoso pos-Operatorio (TREVO)—Risco e mortalidade por especialidade cirurgica). Rev. Port. Cardiol..

[30] Alvin M.D., Alentado V.J., Lubelski D., Benzel E.C., Mroz T.E. (2018). Cervical spine surgery for tandem spinal stenosis: The impact on low back pain. Clin. Neurol. Neurosurg..

[31] Inoue T., Ando K., Kobayashi K., Nakashima H., Ito K., Katayama Y., Machino M., Kanbara S., Ito S., Yamaguchi H. (2021). Primary cervical decompression surgery may improve lumbar symptoms in patients with tandem spinal stenosis. Eur. Spine J..

