# Supplementary material for: Perioperative Outcomes Following Single-Stage Surgery for Tandem Spinal Stenosis—A Single-Center Retrospective Cohort
Source: J Pers Med. 2026 Jun 26;16(7):347. doi: 10.3390/jpm16070347 (PMC13412183; doi:10.3390/jpm16070347)
Supplement: Supplementary file 1 [file jpm-16-00347-s001.zip › jpm-4338024-supplementary.pdf]

Variable	Degenerative	Traumatic	Lesion-related
Patients, n (%)	16 (80.0%)	2 (10.0%)	2 (10.0%)
Total OR time, mean (SD), min	309.6 (90.5)	422.5 (71.4)	477.5 (279.3)
Operative time, mean (SD), min	214.4 (92.1)	286.5 (10.6)	313.5 (176.1)
Estimated blood loss, mean (SD), mL	208.4 (254.4)	275.0 (176.8)	400.0 (0.0)
Hospital length of stay, mean (SD), days	4.7 (2.0)	24.2 (10.9)	7.3 (4.2)

Table S1. Descriptive perioperative outcomes stratified by etiologic subgroup

Degenerative includes stenosis, disc disease, spondylosis, cauda equina attributed to stenosis, and non-traumatic spinal cord compression; traumatic includes vehicle-accident cases; lesion-related includes intradural extramedullary tumor and thoracic cyst cases.
